# Older adults’ compliance with mobile ecological momentary assessments in behavioral nutrition and physical activity research: pooled results of four intensive longitudinal studies and recommendations for future research

**DOI:** 10.1186/s12966-024-01629-z

**Published:** 2024-08-26

**Authors:** Sofie Compernolle, T. Vetrovsky, I. Maes, J. Delobelle, E. Lebuf, F. De Vylder, K. Cnudde, J. Van Cauwenberg, L. Poppe, D. Van Dyck

**Affiliations:** 1 Department of Movement and Sport Sciences, Faculty of Medicine and Health Sciences, Ghent University, Watersportlaan 2 Ghent, Ghent, B-9000 Belgium; 2 Research Foundation Flanders (FWO), Brussels, Belgium; 3 Faculty of Physical Education and Sport, Charles University, Prague, Czech Republic; 4 Department of Public Health and Primary Care, Faculty of Medicine and Health Sciences, Ghent University, Ghent, Belgium

**Keywords:** Experience sampling, Compliance, Non-response, Ecological momentary assessment

## Abstract

**Background:**

Mobile Ecological Momentary Assessment (EMA) is increasingly used to gather intensive, longitudinal data on behavioral nutrition, physical activity and sedentary behavior and their underlying determinants. However, a relevant concern is the risk of non-random non-compliance with mobile EMA protocols, especially in older adults. This study aimed to examine older adults’ compliance with mobile EMA in health behavior studies according to participant characteristics, and prompt timing, and to provide recommendations for future EMA research.

**Methods:**

Data of four intensive longitudinal observational studies employing mobile EMA to understand health behavior, involving 271 community-dwelling older adults (M = 71.8 years, SD = 6.8; 52% female) in Flanders, were pooled. EMA questionnaires were prompted by a smartphone application during specific time slots or events. Data on compliance (i.e. information whether a participant answered at least one item following the prompt), time slot (morning, afternoon or evening) and day (week or weekend day) of each prompt were extracted from the EMA applications. Participant characteristics, including demographics, body mass index, and smartphone ownership, were collected via self-report. Descriptive statistics of compliance were computed, and logistic mixed models were run to examine inter- and intrapersonal variability in compliance.

**Results:**

EMA compliance averaged 77.5%, varying from 70.0 to 86.1% across studies. Compliance differed among subgroups and throughout the day. Age was associated with lower compliance (OR = 0.96, 95%CI = 0.93–0.99), while marital/cohabiting status and smartphone ownership were associated with higher compliance (OR = 1.83, 95%CI = 1.21–2.77, and OR = 4.43, 95%CI = 2.22–8.83, respectively). Compliance was lower in the evening than in the morning (OR = 0.82, 95%CI = 0.69–0.97), indicating non-random patterns that could impact study validity.

**Conclusions:**

The findings of this study shed light on the complexities surrounding compliance with mobile EMA protocols among older adults in health behavior studies. Our analysis revealed that non-compliance within our pooled dataset was not completely random. This non-randomness could introduce bias into study findings, potentially compromising the validity of research findings. To address these challenges, we recommend adopting tailored approaches that take into account individual characteristics and temporal dynamics. Additionally, the utilization of Directed Acyclic Graphs, and advanced statistical techniques can help mitigate the impact of non-compliance on study validity.

**Supplementary Information:**

The online version contains supplementary material available at 10.1186/s12966-024-01629-z.

## Introduction

Ecological Momentary Assessment (EMA), also known as Experience Sampling Method, has gained increasing popularity for collecting intensive, longitudinal data on behavioral nutrition, physical activity and sedentary behavior and their underlying determinants [1, 2]. During EMA, participants are repeatedly prompted to self-report on their real-time behaviors, experiences and contexts using paper-based diaries or electronic devices, such as smartphones. This prompting can occur at random moments within predetermined intervals throughout the day, constituting the time-based sampling approach, or when specific events take place, such as consuming a snack, or engaging in prolonged sedentary behavior, known as event-based sampling [3, 4]. One of the main advantages of EMA is its reduced susceptibility to recall bias, making it more accurate than retrospective methods, especially for populations often dealing with recall problems (e.g., older adults) [4, 5]. Moreover, because of the time-intensive nature of the collected data, typically involving multiple data points per day, EMA has the potential to shed light on patterns and fluctuations in behaviors, along with the factors that influence these variations. This temporal information allows for a deeper understanding of how health behaviors and determinants evolve over time. Finally, by observing behaviors and experiences in naturalistic settings, EMA yields ecologically relevant data [6].

All these advantages make EMA a promising data collection method for health behavior research; however, low compliance with EMA protocols might be an important concern [7]. Note that, in this paper, compliance is referred to as the extent to which participants responded to the EMA prompts by filling out one or more questions. Low compliance in EMA studies can be expected due to the substantial burden that is placed on participants when asked to repeatedly complete a number of assessments [7]. High levels of non-compliance might significantly affect the validity of EMA results. In the past, several researchers have attempted to quantify compliance with EMA protocols through meta-analyses [8–11]. For instance, Jones et al. revealed a pooled compliance rate of 75% in EMA studies assessing the use of substances of abuse (alcohol, nicotine, cannabis, etc.) among substance users [8], while Williams et al. found a pooled compliance rate of 81% in EMA studies measuring health-related behaviors and perceptual experiences including symptoms, affect or mood in adults [9]. The highest compliance rate was reported by Yao et al. who summarized EMA studies conducted in older adults [11]. Contrary to prevailing expectations, based on smartphone use and digital literacy [12], their meta-analysis revealed a surprisingly high pooled compliance rate of 86% [11].

It is however important to highlight the heterogeneity observed across individual studies included in the meta-analyses. This heterogeneity might stem from differing approaches in data analysis; while some studies chose to exclude participants with low compliance, others did not [11]. Also, design characteristics might have contributed to the differences in compliance rate. While certain meta-analyses showed evidence of an association between design characteristics, including the frequency of prompts per day and the items per prompt, and compliance, others did not find such correlations [8–10]. The only design factor that was consistently (positively) correlated with compliance was the provision of incentives [10]. Lastly, the diversity in study populations might have contributed to the observed heterogeneity in compliance rates. Unfortunately, there is a dearth of information on the characteristics distinguishing individuals with low compliance rates from those with high compliance rates, especially in older adults. However, it is possible that certain subgroups of older adults are more prone to non-compliance compared to others —for example, due to unfamiliarity with continuously monitoring their smartphones. Insights into individual characteristics contributing to varying levels of older adults’ compliance could inform the design of future EMA protocols. Furthermore, little is known about how the compliance of individuals varies within and between days and how it evolves over time. Thus, it remains unclear whether non-compliance occurs randomly or follows specific patterns. Understanding this distinction is crucial, as the presence of non-random non-compliance is detrimental to the validity of the EMA results [13].

Patterns of non-compliance or missing data can be categorized as ‘missing completely at random’ (MCAR), ‘missing at random’ (MAR), or ‘missing not at random’ (MNAR) [14]. In the case of MCAR, we assume that the probability of missingness is unrelated to both observed and unobserved data, implying that the absence of data follows no discernible pattern. This would be the case when the participant did not respond to an EMA questionnaire because they have missed the trigger due to not having their smartphone with them. For MAR, we assume that the likelihood of missingness is related to observed data, such as socio-demographics at baseline, but not to unobserved data. For instance, men might be less inclined to respond to an EMA questionnaire on physical activity compared to women. As leisure-time physical activity tends to be higher in men than in women [15], the general prevalence of leisure-time physical activity might then be underestimated. Statistical techniques, such as multiple imputation and inverse probability weighting, can be employed to mitigate this issue. The most challenging scenario arises with MNAR, where the probability of missingness is associated with the unobserved data, or in this case the missing EMA data, themselves. For example, if individuals only report instances of snacking when their snack was healthy, the study results will obviously be biased. Detecting and addressing MNAR poses considerable challenges in EMA research, and corrective measures may not be sufficient, depending on the extent and nature of the missing data [16].

The aim of this study was to examine older adults’ compliance with EMA protocols in detail. Firstly, we described older adults’ compliance with EMA (research question 1). Secondly, we investigated interpersonal variability in older adults’ compliance with EMA by examining the role of individual characteristics, including socio-demographics, body mass index (BMI) and smartphone ownership (research question 2). Thirdly, we estimated intrapersonal variability in older adults’ compliance by examining variances in compliance within days, and between week and weekend days (research question 3). Lastly, we combined the second and the third research questions to examine if individual characteristics significantly contribute to the intrapersonal variability in levels of compliance (research question 4). By doing so, in-depth information was gathered on the mechanisms underlying missingness of older adults’ compliance with EMA protocols, and recommendations were formulated for the design of future EMA studies focused on older adults.

## Methods

### Study design

This paper reports on the results of four intensive longitudinal observational studies with EMA conducted between November 2019 and October 2022 in Flemish community-dwelling older adults with a combined sample of 271 participants. Study reporting was conducted according to the STROBE checklist (see Additional file 1). Study 1 explored the time-dependent variability of emotions, physical complaints, intention, and self-efficacy towards physical activity in a sample of 67 older adults (65+) [17]. Study 2 investigated within-person associations of visibility of snacks, social modeling, intention and emotions with snacking behaviors in a sample of 51 older adults (60+) [18]. Studies 3 and 4 examined the contextual, affective and physical states (i.e., pain and fatigue) of older adults (65+) during physical activity (*n* = 79) and sedentary behavior (*n* = 74), respectively. All studies obtained ethical approval by the Ghent University Hospital Ethics Committee (2019/0192; B6702021000698; BC-09944).

### Participants and procedure

Participants in all four studies were recruited through purposive convenience and snowball sampling to ensure heterogeneity in age, gender and educational level. Recruitment primarily occurred through word-of-mouth in the social network of the involved researchers, advertisements on social media and by contacting community associations for older adults. Older adults were eligible to participate in studies 1, 3 and 4 if they were 65 years or older, and in study 2 if they were 60 years or older. Older adults who had been diagnosed with a cognitive disease, those with severe hearing or vision impairment, and those with insufficient knowledge of the Dutch language were ineligible to participate in all studies. Additionally, those not able to walk 100 m without assistance were also ineligible for study 1, 3 and 4. Owning a smartphone was not included in the eligibility criteria, as those without a smartphone were provided with one by the researchers (Motorola Moto G20, Motorola Moto E40 or Wiko Lenny 3, 64GB, Android 6.0).

All participants were visited at home before the start of the EMA data collection. During this first home visit, study information was provided, informed consents were signed, baseline measures were collected and instructions were given on the EMA data collection. For those who received a smartphone from the researchers, a small introductory training was provided. The EMA data collection lasted seven days in each of the studies. All questionnaires were automatically triggered by a smartphone application (i.e. Smartphone Ecological Momentary Assessment 3 [SEMA^3^] [19] or HealthReact [version 1.62, University of Hradec Králové, Czechia]) during predefined time slots (study 1 and 2), or after specific events (study 3 and 4). The maximum number of automatically triggered questionnaires varied from five to six per day. Detailed information on the EMA data collection of each of the four studies is presented in Table 1, in previous publications [17, 18, 20], and on OSF (https://osf.io/gdzmv/, https://osf.io/djzsf/, https://osf.io/94tcb/).

**Table 1:** EMA data collection characteristics

EMA data collection	Study 1 (physical activity)	Study 2 (snacking)	Study 3 (physical activity)	Study 4 (sedentary behavior)
Study duration	7 days	7 days	7 days	7 days
Sampling strategy	Time-based	Time-based	Event-based (after 5’ of sustained walking)	Event-based (after 30’ of sedentary behaviour)
Sampling frequency	6 triggers/day	5 triggers/day	Depending on the number of walking events; max. of 6 triggers/day	Depending on the number of sedentary events; max. of 6 triggers/day
Minimum sampling interval	30 min	30 min	60 min	120 min
Sampling device and application	Smartphone with SEMA^3^	Smartphone with SEMA^3^	Smartphone with HealthReact (linked to Fitbit)	Smartphone with HealthReact (linked to Fitbit)
Number of items per prompt	15 items (evaluated on a 7 point Likert scale)	10 items (evaluated on a 7 point Likert scale)	1 to 12 items (depending on their answers)	1 to 14 items (depending on their answers)
Number of reminders	Two reminders (after 5’ and 10’)	Two reminders (after 5’ and 10’)	Seven reminders (after 10’, 20’, 25’, 26’, 27’, 28’, 29’)	Seven reminders (after 10’, 20’, 25’, 26’, 27’, 28’, 29’)
Delay allowed to answer the survey*	20 min	20 min	30 min	30 min

* “Delay allowed to answer the survey” indicates the duration for which the survey remains accessible after being prompted

### Measures

Participant’s characteristics were collected during the baseline visit and included: age, gender, educational level, main occupation (prior to retirement), marital status, height, weight and smartphone ownership. Height and weight were used to calculate participants’ BMI. Time and day of each prompt and information whether a participant answered at least one item following the prompt (i.e. compliance) were extracted from the EMA applications. Time was coded as morning (6:00 am – 11:59 am), afternoon (12:00 pm – 17:59 pm) or evening (18:00 pm – 23:59 pm), and day was coded as weekday or weekend day.

### Data analysis

Descriptive statistics of compliance were computed and plotted per day for the whole sample and per study (research question 1). Four-level logistic mixed models with compliance as a binary outcome variable were used to answer research question 2–4. Random intercepts were included at the study, person, and day levels to account for the nested structure of the data (i.e., EMA questionnaires were nested within days, days were nested in individuals and individuals were nested in studies), and the respective variances were calculated. Between- and within-person variances in older adults’ compliance with EMA questionnaires were estimated using a four-level unconditional random intercept logistic model. The percentage of variance attributable to a specific level (study, person, day and within-day) was calculated by dividing the variance components of the grouping variables by the total variance (i.e., sum of the variance components), and the likelihood ratio test was used to determine the statistical significance of the variance components as specified by Snijders and Bosker [21].

Socio-demographic factors (age, gender, educational level, marital status, and main occupation [prior to retirement]), BMI, and smartphone ownership were individually added as explanatory variables in the four-level logistic mixed model to explore their associations with EMA compliance (research question 2). Subsequently, two additional explanatory variables, namely, day of the week (coded as weekday = 0; weekend day = 1) and time of the day (coded as 6:00 am – 11:59 am = 0; 12:00 pm – 17:59 pm = 1; 18:00 pm – 23:59 pm = 2), were separately added to the four-level logistic mixed model (research question 3).

Finally, a set of additional logistic mixed models examining the moderating effects of socio-demographic factors, and BMI on the associations of day of the week and time of the day with EMA compliance were conducted by adding two-way interaction terms to the four-level logistic mixed model. Chi^2^-tests were applied to the results of the final set of logistic mixed models when the interaction terms contained a nominal variable with more than two categories (research question 4).

## Results

### Sample characteristics

Table 2 presents the descriptive statistics of the total sample (*n* = 271) and each of the study samples. The total sample had a mean age of 71.8 years (± 6.8). Participants in studies 1, 3, and 4 shared a comparable average age, while the mean age of participants in study 2 was relatively younger. Gender was equally distributed in the total sample and each level of educational attainment was well represented. The majority of the sample was married or cohabiting (75.3%) and fell into the white-collar occupational category (57.4%). Mean BMI was 25.7 kg/m^2^ (± 3.8), and 88.1% of the sample had a smartphone.

**Table 2:** Sample characteristics and EMA compliance

Characteristics	Total sample (*n* = 271)	Study 1 (*n* = 67)	Study 2 (*n* = 51)	Study 3 (*n* = 79)	Study 4 (*n* = 74)	F or χ2	*P*-value
Age (years; mean ± SD; and range)	71.8 (± 6.8) 60–92	72.3 (± 6.0) 65–86	66.2 (± 6.2) 60–85	73.0 (± 6.8) 64–92	74.0 (± 5.9) 65–87	18.0	< 0.001
Gender						3.3	0.34
Men (%)	48.0%	53.7%	47.1%	51.9%	39.4%		
Education						25.2	0.05
Lower (%) Middle (%) Higher (%)	19.9% 43.6% 36.5%	25.4% 34.3% 40.3%	31.4% 41.2% 27.5%	18.2% 49.4% 32.5%	8.6% 47.1% 44.3%		
Marital status						18.7	0.03
Married or cohabiting (%)	75.3%	85.1%	82.4%	72.7%	63.4%		
Occupation						41.0	0.09
Household (%) Blue collar (%) White collar (%) Other (%)*	9.1% 30.0% 57.4% 3.4%	4.5% 37.3% 52.2% 6.0%	3.9% 19.6% 72.5% 3.9%	16.2% 33.8% 48.6% 1.4%	10.0% 25.7% 61.4% 2.9%		
BMI (kg/m^2^; mean ± SD) Underweight (%) Normal weight (%) Overweight (%) Obese (%)	25.7 (± 3.8) 2.6 40.4 42.7 14.2	25.6 (± 4.0) 6.0 41.8 37.3 14.9	25.2 (± 3.6) 2.0 45.1 43.1 9.8	26.3 (± 3.8) 1.3 34.6 46.2 17.9	25.7 (± 3.7) 1.4 42.3 43.7 12.7	0.95 7.2	0.42 0.62
Smartphone ownership (%)	88.1%	83.6%	94.1%	NA	NA	NA	

SD = standard deviation; Lower education = Non-secondary education, Middle education = Non-tertiary education, Higher education = Tertiary education; NA = not available; *the category ‘Other’ comprised occupations that could not be categorized due to the lack of information; Analysis of Variance and Chi^2^-tests were conducted to examine differences between study samples

### Compliance with EMA (research question 1)

**Fig. 1:**
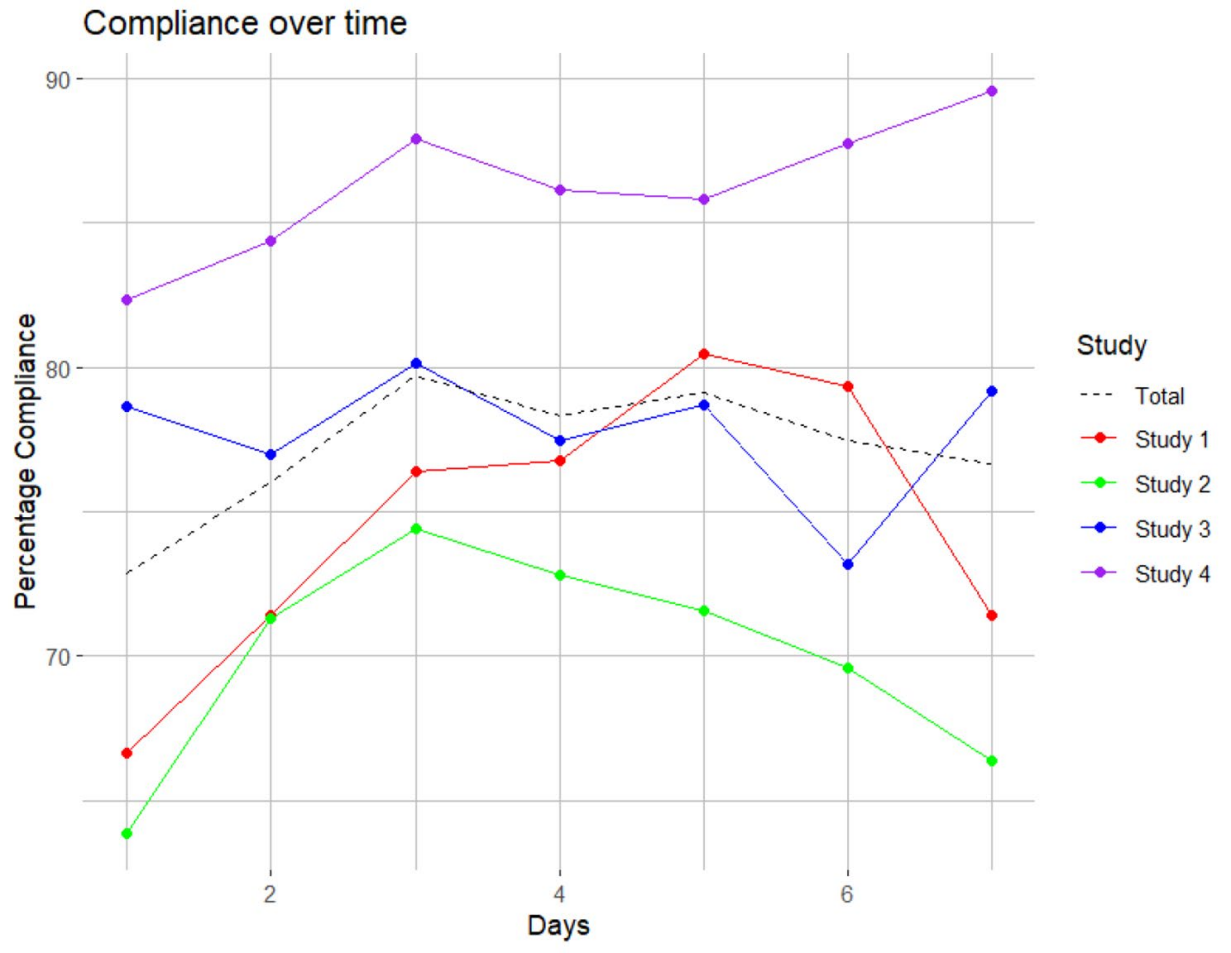
Temporal patterns of compliance with EMA

Figure 1 illustrates the temporal pattern of compliance with EMA during the study week across the entire sample (depicted by the black dashed line), as well as individual study samples (represented by colored lines). The overall average compliance with the EMA was 77.5%, with variations ranging from 70.0% in study 2 to 86.1% in study 4. A clear increase in compliance is visible from the first study day to the second and the third study day, except for study 3, where compliance levels remained relatively constant. In three out of four studies, the lowest compliance occurred on the first day of the study, while peak compliance varied considerably between the studies.

### Inter- and intrapersonal variability of older adults’ compliance with EMA (research question 2 and 3)

Table 3 presents the random and fixed effects of the logistic mixed models of compliance with EMA in older adults. The random part of the four-level unconditional random intercept logistic mixed model showed that most of the variance in compliance was due to between-person differences within studies (91.40%), followed by between-day within-person differences (6.99%) and between-study differences (1.61%). None of the variance in compliance was explained by within-day within-person differences, or in other words by differences between the different EMA questionnaires of a person on the same day. The fixed part of the four-level logistics models showed that the odds of compliance decreased by an average of 4% with each additional year of age (OR = 0.96, 95% CI = 0.93, 0.99). Participants who were married or cohabiting had on average a 83% higher odds of compliance compared to those who lived alone (OR = 1.83, 95% CI = 1.21, 2.77). Gender, occupation, educational level and BMI were not significantly related to the odds of compliance. The odds of compliance for participants who owned a smartphone was on average 4.43 times higher compared to those without a smartphone (OR = 4.41, 95% CI = 2.22, 8.83). EMA questionnaires that were sent in the evening had on average a 18% lower odds to be responded to than those that were sent in the morning (OR = 0.82, 95%CI = 0.69, 0.97). Weekday vs. weekend day was not significantly associated with the odds of compliance.

**Table 3:** Random and fixed effects on older adults’ compliance with EMA

Random part	Unconditional random intercepts logistic mixed model		
	Variance (SD)		% total variance
Study-level variance	0.13 (0.36)		6.99%
Person-level variance	1.70 (1.30)		91.40%
Day-level variance	0.03 (0.18)		1.61%
Within-day-level variance	0.00 (0.04)		0.00%
Likelihood Ratio test	χ2 (3) = 1179.50; *p* < 0.001		
Type of Likelihood Ratio test	Random effects		
**Fixed part**	**Four-level logistic mixed models**		
**Interpersonal factors**	**OR**	**p**	**95% CI**
**Socio-demographic factors**			
Age (years)	0.96	0.01	0.93, 0.99
Gender (ref: men)			
Women	1.08	0.68	0.75, 1.54
Educational level (ref: high)			
Low Middle	1.03 0.93	0.92 0.72	0.62, 1.71 0.64, 1.38
Marital status (ref: alone)			
Married or cohabiting	1.83	< 0.01	1.21, 2.77
Occupation (ref: white-collar)			
Blue-collar Household Other	0.47 1.43 0.56	0.05 0.29 0.22	0.45, 1.00 0.34, 1.32 0.27, 1.71
**BMI (kg/m** ^**2**^ **) – continuous**	0.97	0.22	0.93, 1.02
**Smartphone ownership (ref: no)**	4.43	< 0.001	2.22, 8.83
**Intrapersonal factors**			
Day of the week (ref: weekday)			
Weekend day	0.89	0.09	0.78, 1.02
Period of the day (ref: morning)			
Afternoon Evening	1.04 0.82	0.58 0.02	0.90, 1.20 0.69, 0.97

OR = odds ratio; CI = confidence interval; ref = reference

### Moderating effects of socio-demographic factors and BMI in the intrapersonal variability of older adults’ compliance with EMA (research question 4)

**Fig. 2:**
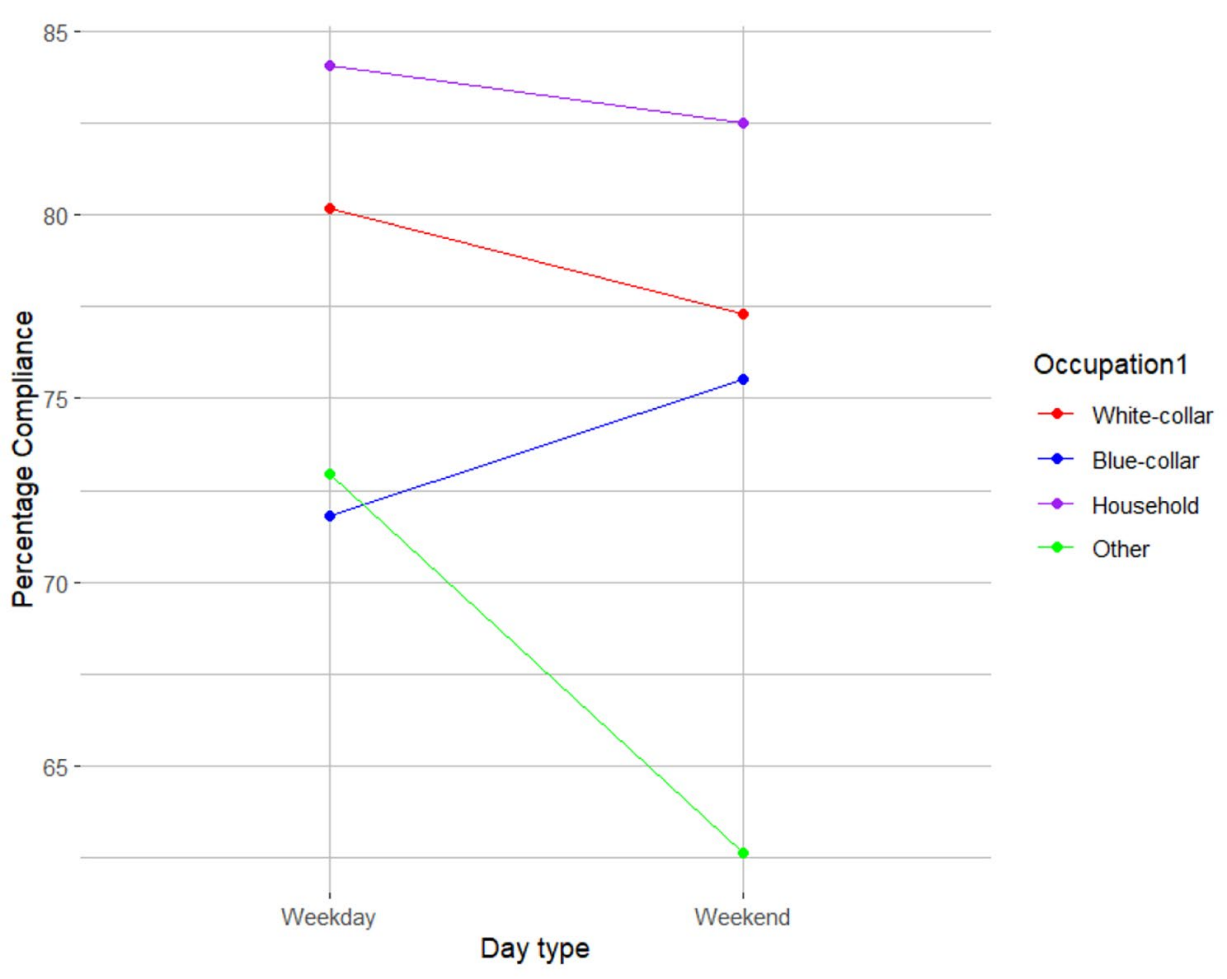
Compliance with EMA between participants from different occupational categories by type of the day

The variation in compliance with EMA across different times of the day was not moderated by socio-demographic factors. However, the difference in compliance with EMA between week and weekend days was moderated by occupational status before retirement (χ^2^ = 9.22; *p* = 0.03). Whereas those with a blue-collar occupation before retirement exhibited the highest compliance on weekend days, all other participants demonstrated highest compliance on weekdays (Table 4; Fig. 2).

**Table 4:** Interaction effects of time of day and day of week on compliance with EMA

Interaction terms	χ2*	*P*	Interaction terms	OR or χ2*	95% CI or *p*
Time of the day by Age	3.42	0.18	Day of the week by Age	0.97	0.74,1.26
Time of the day by Gender	4.78	0.09	Day of the week by Gender	0.98	0.75, 1.27
Time of the day by Educational level	7.46	0.11	Day of the week by Educational level	3.97	0.14
Time of the day by Marital status	3.21	0.20	Day of the week by Marital status	0.83	0.61,1.14
Time of the day by Occupational status	5.12	0.53	Day of the week by Occupational status	9.22	0.03
Time of the day by BMI	4.08	0.13	Day of the week by BMI	1.10	0.84, 1.43

* Chi^2^-tests were applied to the results of the final set of logistic mixed models when the interaction terms contain a nominal variable with more than two categories

## Discussion

This study aimed to examine older adults’ compliance with EMA protocols, focusing on the impact of both inter- and intrapersonal variables. The findings provide valuable insights into the patterns associated with EMA compliance in this population and highlight some critical issues and challenges that should be considered when designing future EMA studies among older adults.

The average compliance rate of 77% across the entire sample is consistent with previous research in older adults [11]. However, in study 2, compliance was notably lower, possibly due to the lower age limit of 60 years, differing from the other three studies where the lower limit was set at 65 years. This variation in age limit may have led to a sample including individuals who are not yet retired. While our results suggested that the odds of compliance decrease with age, this trend may only apply to retired older adults. Another factor to consider is the focus of the research. Study 2 was the only study concentrating on dietary behavior. It might be that older adults are less inclined to respond to EMA questions related to dietary habits compared to physical activity and sedentary behavior. As the current study was not designed to examine the influence of study topic on compliance, future research should explore this possibility further. The observed increase in compliance over the first study days, except in study 3, suggests improved engagement as participants become more familiar with the assessment process. Future researchers might consider removing data from the first, or first two measurement days, if sufficient data is available, to mitigate the influence of initial adjustment. The notable variability in peak compliance between studies underscores the importance of considering study-specific factors that may influence participant compliance with EMA. Previous reviews have already summarized the study design characteristics that might contribute to compliance with EMA, such as the number of prompts per day and the number of items per prompt [1, 8–11], however, none of the reviews examined peak compliance over time. Although the four intensive longitudinal studies included in the current study shared similar study characteristics, differences in sampling strategy, number of prompts, number of items included in the EMA questionnaire, and target behavior might have affected (peak) compliance. However, examining these differences was outside the scope of the current study.

Based on our pooled results, the highest variance in older adults’ compliance with EMA is attributable to interpersonal factors. Age emerged as a significant predictor of compliance with older participants exhibiting lower odds of compliance. This finding is in line with our expectation based on digital literacy assumptions. The older the elderly get the less likely they have well-developed digital skills, which are crucial to successfully comply with device-based EMA [22, 23]. Furthermore, marital/cohabiting status also emerged as a significant predictor of older adults’ compliance with EMA. The positive association between marital/cohabiting status and compliance suggests a potential role of social support in fostering engagement. A recent systematic review stressed the importance of social support to overcome technical issues and increase motivation in mobile health interventions [24]. Finally, smartphone ownership also emerged as a strong predictor of older adults’ compliance with EMA. This underscores the importance of access to and familiarity with technology in facilitating engagement and adherence to EMA procedures. Although a small introductory training was provided for those without a smartphone in the current studies, our findings suggest that this may not adequately prepare participants to consistently comply with the EMA protocol. Hence, future research should carefully consider the inclusion of older adults without smartphones in EMA studies and should provide sufficient familiarization or training before the start of the study.

Furthermore, our study shed light on the impact of intrapersonal factors on older adults’ compliance with EMA. Although their role appeared relatively small compared to interpersonal factors, we observed that participants exhibited markedly lower levels of compliance in the evening compared to the morning or afternoon. This was not in line with previous research, conducted in children, adolescents and adults, showing higher levels of compliance in the evening [10, 25], and suggests that temporal fluctuations in motivation, activities, contexts or other intrapersonal variables might have influenced the willingness or opportunity of older adults to comply with EMA requirements [13, 26]. Understanding the temporal fluctuations in compliance provides an opportunity to optimize EMA data collection strategies.

Based on the current results, it is evident that non-compliance in our pooled dataset is not completely at random. As outlined in the introduction, non-compliance and missing data can be categorized into three distinct types: MCAR, MAR, and MNAR. Given that specific population subgroups, such as the oldest older adults, those living alone, and those without a smartphone, exhibit a higher likelihood for non-compliance, and that non-compliance tends to be more prevalent in the evening, it appears that the tendency for missingness is at best associated with observed data, aligning with the categorization of MAR. The presence of MAR data could introduce bias, potentially compromising both the internal and external validity of study outcomes, if not properly addressed [13, 27]. For instance, consider a scenario where one aims to describe the sedentary activities of older adults; activities typically occurring in the evening might be underrepresented. Similarly, when examining the social contexts of physical activities in older adults, activities usually performed by participants living alone may be inadequately represented. Therefore, overlooking or inadequately addressing non-compliance with EMA could indeed present a significant threat.

To improve the validity of future EMA studies in older adults, several mitigation strategies can be implemented, contingent upon the nature of the research question being addressed. For descriptive or predictive research questions, baseline oversampling of respondents with specific characteristics, or providing tailored protocols to improve their compliance may suffice [28]. These tailored protocols may include strategically scheduled assessments during periods when older adults are more likely to be receptive and attentive, or strategies that directly address the challenges or motivations associated with evening assessments. For causal research questions, we recommend drawing directed acyclic graphs (DAGs) during the study design phase [29–31]. DAGs serve as powerful visual tools, developed based on expert knowledge, about the hypothesized causal relationships between the various factors influencing compliance and can be used to identify selection/collider bias (i.e. the change in the association between an exposure and an outcome under study when conditioning on [via restriction, stratification or regression adjustment] a collider [i.e. a third factor influenced by both exposure and outcome]) [29, 31, 32]. Consider a study investigating the causal effect of emotions on snacking behavior. Within this study, compliance could act as a collider because it is likely to be affected by emotions as well as snacking behavior (i.e., participants not reporting unhealthy snacking because of social desirability). Selecting only those who comply with the EMA questionnaire, is the same as conditioning on the collider, potentially yielding biased estimates. By proactively identifying sources of bias through DAGs and applying mitigation strategies in the study design, researchers can enhance the validity of their EMA findings. Finally, the integration of statistical techniques like multiple imputation or inverse probability weighting should be applied in the case of MAR data patterns [33, 34]. Multiple imputation is a statistical approach employed to deal with missing data by imputing several sets of plausible values for each missing observation. These imputed datasets are then analyzed separately, and the results are combined using specialized methods that account for the variability introduced by the imputation process, providing more robust estimates. Inverse probability weighting is a statistical technique used to adjust for selection bias in observational studies. It involves assigning weights to each observation based on the inverse of the probability of being sampled or included in the study, thereby giving more weight to observations that are less likely to be selected. Both statistical methods offer robust approaches to handle missing data, thereby enhancing the reliability of study findings.

### Strengths and limitations

This study is the first to combine EMA datasets from a diverse range of health behavior studies to gain in-depth insight into older adults’ compliance with EMA. By pooling the data, we were able to conduct the analysis on a large dataset, thereby assuring sufficient statistical power to examine intra- and interpersonal variability in older adults’ compliance with EMA. The recommendations formulated in this study will provide valuable guidance for researchers in developing more effective EMA protocols tailored to the unique needs and characteristics of older adult populations.

Important limitations that should be considered when interpreting study findings include firstly, the fact that all four included studies were conducted in Flemish community-dwelling older adults, which may limit the generalizability of the results to other populations of older adults, especially those from different geographical or cultural backgrounds. Replicating similar studies in diverse settings and populations would be crucial to ascertain the external validity of the identified factors influencing compliance. Secondly, the study exclusively focused on mobile EMA studies. While the use of mobile EMA has increased significantly in recent years, the results cannot be transferred to alternative methods, including paper diaries or phone call check-ins, which are still widely used to collect EMA data. Future studies could benefit from a more inclusive approach that combines mobile EMA with alternative methods. However, measuring compliance when using alternative methods is much more challenging. Lastly, the study lacks detailed insights into the reasons behind non-compliance – making it impossible to rule out MNAR. Future studies should collect and analyze qualitative information by means of interviews or focus group discussions, such as in the study of Ziesemer et al. [35], or use short recording of people’s surroundings when they did not comply with an EMA, such as in the study of the study of Sun et al. [36] to gain a deeper understanding of the factors contributing to lower compliance in certain groups or during specific times of the day. This supplementary data would provide actionable insights for refining study designs and tailoring interventions to address the unique needs and challenges of specific subgroups to increase compliance.

## Conclusions

In conclusion, this study thoroughly examined older adults’ compliance with EMA protocols. Variability in compliance among subgroups and throughout the day is evident, with age being associated with lower compliance, and marital/cohabiting status and smartphone ownership showing a positive association. Notably, compliance is lower in the evening than in the morning, indicating non-random patterns that could impact internal validity. These findings should be taken into account when developing EMA protocols for older adults. Applying tailored approaches that consider individual characteristics and temporal patterns, along with the implementation of statistical techniques to address MAR data, are recommended to enhance the validity of insights into the complex dynamics of health behaviors in this population.

### Electronic supplementary material

Below is the link to the electronic supplementary material.

Supplementary Material 1

## Data Availability

Data from study 1 are available via the Peerj publication (DOI: 10.7717/peerj.13234/supp-1). Data from study 2–4 are published on OSF (https://osf.io/gdzmv/, https://osf.io/djzsf/, https://osf.io/94tcb/).

## Abbreviations

EMA: Ecological momentary assessment
MCAR: Missing completely at random
MAR: Missing at random
MNAR: Missing not at random
BMI: Body mass index
SEMA: Smartphone ecological momentary assessment
SD: Standard deviation
NA: Not available
OR: Odds ratio
CI: Confidence interval
ref: Reference
